# From *Saccharomyces cerevisiae* to Ethanol: Unlocking the Power of Evolutionary Engineering in Metabolic Engineering Applications

**DOI:** 10.3390/jof9100984

**Published:** 2023-09-29

**Authors:** Alican Topaloğlu, Ömer Esen, Burcu Turanlı-Yıldız, Mevlüt Arslan, Zeynep Petek Çakar

**Affiliations:** 1 Department of Molecular Biology and Genetics, Faculty of Science and Letters, Istanbul Technical University, Istanbul 34469, Türkiye; topaloglual@itu.edu.tr (A.T.); eseno15@itu.edu.tr (Ö.E.); 2 Dr. Orhan Öcalgiray Molecular Biology, Biotechnology and Genetics Research Center (ITU-MOBGAM), Istanbul Technical University, Istanbul 34469, Türkiye; turanli@gmail.com; 3 Department of Genetics, Faculty of Veterinary Medicine, Van Yüzüncü Yıl University, Van 65000, Türkiye; mevlutarslan@yyu.edu.tr

**Keywords:** adaptive laboratory evolution (ALE), bioethanol, biofuel, directed genome evolution, ethanol tolerance, ethanol production, evolutionary engineering, genome editing, metabolic engineering, *Saccharomyces cerevisiae*

## Abstract

Increased human population and the rapid decline of fossil fuels resulted in a global tendency to look for alternative fuel sources. Environmental concerns about fossil fuel combustion led to a sharp move towards renewable and environmentally friendly biofuels. Ethanol has been the primary fossil fuel alternative due to its low carbon emission rates, high octane content and comparatively facile microbial production processes. In parallel to the increased use of bioethanol in various fields such as transportation, heating and power generation, improvements in ethanol production processes turned out to be a global hot topic. Ethanol is by far the leading yeast output amongst a broad spectrum of bio-based industries. Thus, as a well-known platform microorganism and native ethanol producer, baker’s yeast *Saccharomyces cerevisiae* has been the primary subject of interest for both academic and industrial perspectives in terms of enhanced ethanol production processes. Metabolic engineering strategies have been primarily adopted for direct manipulation of genes of interest responsible in mainstreams of ethanol metabolism. To overcome limitations of rational metabolic engineering, an alternative bottom-up strategy called inverse metabolic engineering has been widely used. In this context, evolutionary engineering, also known as adaptive laboratory evolution (ALE), which is based on random mutagenesis and systematic selection, is a powerful strategy to improve bioethanol production of *S. cerevisiae*. In this review, we focus on key examples of metabolic and evolutionary engineering for improved first- and second-generation *S. cerevisiae* bioethanol production processes. We delve into the current state of the field and show that metabolic and evolutionary engineering strategies are intertwined and many metabolically engineered strains for bioethanol production can be further improved by powerful evolutionary engineering strategies. We also discuss potential future directions that involve recent advancements in directed genome evolution, including CRISPR-Cas9 technology.

## 1. Introduction

Global energy demand and environmental safety concerns have led to the pursuit of alternative energy sources that are ecologically sustainable. As the global population is increasing, fossil fuel consumption is also increasing, leading to higher emissions of greenhouse gasses and global warming, causing climate change, biodiversity loss and rising sea levels [1]. Biofuels have emerged as an alternative energy source to fossil fuels to secure access to energy and mitigate climate change. Bioethanol is one of the most prominent biofuels, as it burns more efficiently and emits lower greenhouse gases than gasoline, due to its higher evaporation enthalpy and laminar flame speed [2]. 

The first-generation bioethanol is produced by utilizing conventional feedstocks such as glucose derived from starch and sucrose derived from sugarbeet or sugarcane [3]. The second-generation bioethanol, however, is produced by utilizing lignocellulosic biomass from hardwood, softwood and agricultural residues. Thus, lignocellulosic biomass (second generation) represents an alternative feedstock to sugar-starch-containing raw materials (first generation) for bioethanol production due to its low cost, availability, and wide distribution. In addition, the use of lignocellulosic agricultural wastes instead of food and feed crops for bioethanol production is more sustainable, considering the global limitation in both food and energy resources [4]. Lignocellulosic biomass is mainly composed of cellulose (40–60%), hemicellulose (20–40%), and lignin (10–25%) [5]. Hydrolysis of lignocellulosic biomass yields hexose sugars such as glucose, mainly derived from cellulose; and pentose sugars such as xylose and arabinose which are derived from hemicellulose [6]. As reviewed previously, microorganisms that can grow and efficiently utilize pentose sugars are highly desirable for lignocellulosic bioethanol production [7].

The yeast *Saccharomyces cerevisiae* is the most widely used organism for industrial ethanol production, because of its rapid growth, high-efficiency in ethanol yield, and high tolerance to environmental stressors such as ethanol, low pH, and low oxygen. Furthermore, it has the ‘Generally Recognized as Safe’ (GRAS) status [8]. During industrial-scale ethanol production, *S. cerevisiae* has a yield higher than 90% of the theoretical maximum which is approximately 0.51 g ethanol per g of consumed glucose [9]. Hence, even a slight increase in ethanol yield can provide hundreds of millions of dollars of additional profits annually [10].

Metabolic engineering is a scientific discipline that aims to enhance cellular functions and characteristics by modifying the enzymatic, transport, and regulatory functions of the cell, using recombinant DNA technology [11]. Metabolic engineering strategies are widely used for the improvement of *S. cerevisiae* strains to achieve higher ethanol yields. Rational or classical metabolic engineering, which is a top-down approach, involves making certain genetic changes to an organism’s biochemical response network, which requires detailed knowledge of the genetics and biochemistry of the metabolic pathways under consideration [12]. The inverse metabolic engineering strategy, which is a bottom-up approach, has been introduced to avoid such limitations of rational metabolic engineering, and thereby allows improvement of microbial cells without extensive prior knowledge about their metabolic pathways [12]. 

Evolutionary engineering, also called adaptive laboratory evolution (ALE), is an inverse metabolic engineering approach and a potent strategy for obtaining industrially important and desired microbial phenotypes [7]. This approach involves continuous evolution procedures that include a systematic selection method to obtain a desired microbial phenotype [13,14]. Evolutionary engineering has been widely used as a powerful strategy to improve the ethanol yield and productivity of not only wild-type, but also recombinant *S. cerevisiae* strains that have been previously obtained by rational metabolic engineering, as they may usually have low yield and productivity [15,16,17]. 

In this review, following a brief overview of the factors that affect yeast metabolism in bioethanol production and the key genetic techniques used in metabolic engineering applications, we focus on various examples of both metabolic and evolutionary engineering strategies for improved first- and second-generation bioethanol production in *S. cerevisiae*. Many of these examples point out the importance of evolutionary engineering to further improve metabolically engineered *S. cerevisiae* strains for bioethanol production, indicating that metabolic and evolutionary engineering strategies are intertwined. We also discuss potential future directions, including recent developments in directed genome evolution strategies, including CRISPR-Cas9, the state-of-the-art genome editing technology.

## 2. Factors Affecting Yeast Metabolism in Bioethanol Production

The baker’s yeast *S. cerevisiae* is able to rapidly convert sugars into ethanol, which makes it a famous microorganism in bioethanol production. This feature is related to its strong fermentative metabolism. The preferred sugar for *S. cerevisiae* is glucose, a hexose sugar. Glucose is metabolized by the well-known glycolytic pathway, in which one molecule of glucose is converted to two molecules of pyruvate. Under anaerobic conditions, pyruvate is converted in the fermentative pathway of *S. cerevisiae* to acetaldehyde and carbon dioxide, in a reaction catalyzed by the enzyme pyruvate decarboxylase [18]. Acetaldehyde is then reduced to ethanol in the next reaction catalyzed by the enzyme alcohol dehydrogenase I [19].

When edible feedstock is used for first-generation bioethanol production, yeast can utilize various sugars to ferment and produce ethanol, where glucose and the glycolytic pathway are central. However, when the lignocellulosic feedstock is used to produce second-generation ethanol, the utilization of xylose, a pentose sugar, becomes critical for the process efficiency. In lignocellulosic hydrolysates, glucose and xylose are present at 60–70% and 30–40% in all sugar compositions, respectively [20]. However, the yeast *S. cerevisiae* cannot naturally utilize xylose for ethanol fermentation. Thus, *S. cerevisiae* strains that can efficiently utilize xylose are highly demanded for second-generation ethanol production processes.

Yeast metabolism and bioethanol production are affected by several factors. Fermentation temperature, sugar concentration, pH, agitation rate, inoculum size, and fermentation time are the well-known factors [8]. Non-optimal fermentation temperatures limit cell growth and become a stress factor for cells [21]. Thus, careful regulation of fermentation temperature is crucial. For bioethanol production by using yeast, the ideal temperature is between 20 °C and 35 °C, and *S. cerevisiae* has an optimum temperature of about 30 °C as free cells, while this value is slightly higher when the cells are immobilized [22]. Increasing sugar concentration can increase the fermentation rate and bioethanol yield. High sugar contents can lead to steady fermentation rates, as the yeast cells have a high sugar uptake capacity. Very high gravity (VHG) fermentation uses high concentration of sugars during fermentation, which results in increased concentrations of ethanol [23,24]. In this technology, growth of the microorganism is prolonged and enhanced in the presence of low-level oxygen [25]. Since the transportation of some nutrients into yeast cells is affected by H^+^ concentration in the fermentation broth, pH also affects ethanol production. Decrease in yeast growth, bacterial contamination and by-product formation can occur depending on the changes in pH, which may result in lower bioethanol production [8]. The optimum pH range of *S. cerevisiae* is between 4.0–5.0 during bioethanol production by fermentation [26]. Agitation rate is also an important factor for the efficient transport of nutrients from the extracellular environment (culture medium) to the cells and for the transport of the produced ethanol to the extracellular environment. Furthermore, agitation improves mass and heat transfer, and dissolution and dispersion of oxygen through the culture medium, which affects a variety of metabolic activities, including nitrogen assimilation and sterol synthesis. In enology, the increase in yeast nitrogen assimilation by agitation and dissolution of oxygen has been reported previously [27,28,29,30,31]. The commonly used agitation rate for fermentation by yeast cells is in the range of 150–200 rpm [8]. Inoculum size usually has a minor effect on ethanol metabolism and yield. However, it is also an important factor that affects the overall productivity of bioethanol production as it significantly affects fermentation time [32,33]. It has been reported that increasing inoculum concentration from 3.0% to 6.0% resulted in a decrease of 24 h in fermentation time of *S. cerevisiae* [34].

Apart from the above-mentioned factors that affect yeast metabolism and bioethanol production, additional factors associated with the feedstock used during bioethanol fermentation are also important. During second-generation bioethanol production, the lignocellulosic feedstocks need to be pre-treated to make their carbohydrates accessible for enzymatic hydrolysis and ethanol fermentation. Various pre-treatment methods exist, such as acid hydrolysis, steaming/steam explosion (STEX), ammonia freeze explosion (AFEX), and wet oxidation (WO). Acid hydrolysis generates acetic acid from the acetyl group of lignin and hemicellulose at a high rate, ranging from 1 g/L to 15 g/L. The high amount of acetic acid acts as a strong inhibitor of bioethanol production [35]. Lignocellulosic hydrolysates can contain diverse toxic carbohydrate degradation products, including organic acids (e.g., acetic acid), soluble lignin derivatives (phenolics), dehydrated sugar monomers (furans), and 5-hydroxymethylfurfural (HMF) which can significantly inhibit yeast growth and fermentation [36,37,38]. For this reason, yeast strains that are tolerant to such inhibitors are desirable for efficient second-generation bioethanol production.

## 3. Metabolic Engineering of Yeast for Bioethanol Production 

**Figure 1:**
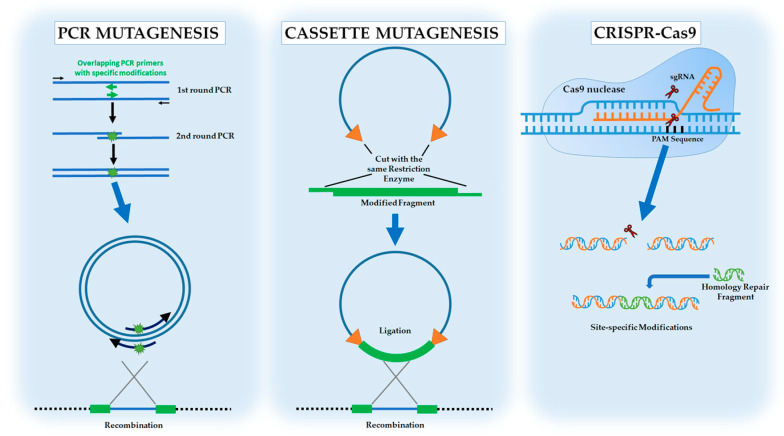
Overview of the key genetic techniques used in metabolic engineering applications, to introduce specific and targeted changes. Using PCR mutagenesis; insertion, deletion or point mutations can be generated. Cassette mutagenesis uses specific restriction sites and modified fragments can be introduced into vectors for further recombination to host organisms. CRISPR-Cas9 is a state-of-the-art genome editing technology in which the modifications can be targeted in a fast and precise fashion.

As mentioned in the Introduction section, rational metabolic engineering requires detailed information on the genetics and biochemistry of the metabolic pathways under consideration and it involves making specific genetic modifications to an organism’s biochemical response network [12]. Since the first introduction of metabolic engineering in 1991 by Bailey as a new scientific discipline [11], there have been significant developments in genetic engineering tools and techniques which enabled researchers to engineer or transfer microbial metabolic pathways with higher efficiency. Specific and targeted changes can be made to a DNA sequence of interest, by a technique known as site-directed mutagenesis that can be utilized for point substitution, deletion, and insertion mutations. Site-directed mutagenesis can be easily performed by using polymerase chain reaction (PCR). As an extensively used technique, the PCR-based gene targeting method includes introduction of exogenous DNA into the host cell by various transformation methods and manipulation of the host genome by the native double-stranded break (DSB) repair system [39]. Another common approach for site-directed mutagenesis is cassette mutagenesis in which a synthetic double-stranded DNA ‘cassette’ containing desired mutations is introduced into a plasmid vector between two restriction sites [40]. However, the major limitation for this approach is the availability of suitable restriction sites that flank the site to be mutated. Most recently, the powerful CRISPR-Cas9 genome editing technology has been extensively used in metabolic engineering research, as discussed in Section 6, Future Directions for Evolution-Based Metabolic Engineering of Yeast for Bioethanol Production. The CRISPR-Cas9 technology uses artificially engineered nucleases to create specific double-stranded breaks at a desired locus, and single or multiple gene editing is achieved by the cell native repair system [39]. Figure 1 shows the key genetic techniques that are used to introduce specific and targeted changes into the host genome for metabolic engineering applications.

### 3.1. Lowering ATP Yield

The first-generation bioethanol production involves utilization of conventional feedstocks like glucose or sucrose [3]. Glucose is catabolized through the Embden–Meyerhof–Parnas (EMP) pathway in *S. cerevisiae* where one mole of glucose is metabolized into two moles of pyruvate and two moles of ATP. The ethanol yield of this pathway is between 90–93%, while the maximal biomass yield is around 7% [41]. ATP is used for growth at the expense of glucose which is not converted to ethanol. Thus, cell growth or biomass formation can be described as a by-product of the first-generation ethanol production. Lowering ATP yield during alcoholic fermentation reduces substrate conversion to biomass and thereby increases ethanol yield [10]. This goal can be achieved using rational metabolic engineering strategies by introducing futile cycles, decreasing the ATP stoichiometry of yeast glycolysis, and modifying the structure and energy coupling of disaccharide metabolism and transport [42].

A metabolic futile cycle occurs when two compounds are interconverted by irreversible reactions and both enzymes catalyzing these reactions are active. There is no change in metabolites, but dissipation of energy (ATP) takes place [43]. Thus, to decrease biomass formation in first-generation bioethanol production by lowering ATP yield, futile cycles can be introduced by rational metabolic engineering strategies, and higher ethanol yields can be achieved [42]. For example, to overcome the tight regulation of phosphofructokinase (PFK) and fructose-1,6-bisphosphatase (FBPase) futile cycle, a bacterial (*Escherichia coli*) FBPase insensitive to fructose-2,6-bisphosphate inhibition was expressed in *S. cerevisiae* which resulted in an increase in ethanol yield by 8.8%, along with an increase in yeast biomass, while decreasing ATP levels by 31–39%, compared to the wild-type strain [44]. Constitutive expression of ATPases may also result in ATP-wasting cycles. In a recent study, 10% increase was achieved in ethanol yield, compared to the parental strain, by the overexpression of the F1 part of the *E. coli* H^+^-ATPase enzyme in *S. cerevisiae.* This also caused a 26% decrease in biomass yield which was overcome by growth-decoupled (nitrogen-starved) conditions with a higher inoculum size that increased volumetric productivity by 111%, compared to the control strain [45]. Semkiv et al. [46] achieved 13% increase in ethanol yield, compared to the parental strain, while declining intracellular ATP level and biomass accumulation by the overexpression of *PHO8* gene in *S. cerevisiae* BY4742 strain. Although the introduction of futile cycles can increase the ethanol yield, it requires careful adjustment to avoid ATP depletion during industrial processes [10].

The ATP stoichiometry of yeast glycolysis can be decreased by introducing the Entner-Doudoroff (ED) pathway of the ethanol-producing bacterium *Zymomonas mobilis* for improved alcoholic fermentation. Instead of 2 moles of ATP generated per mole of glucose by yeast glycolysis, the ED pathway of *Z. mobilis* generates one mole of ATP per mole of glucose with 97% ethanol yield and 3% biomass yield [47]. Benisch and Boles [48] successfully expressed one of the required enzymes of the bacterial ED pathway, KDPG (2-keto-3-deoxy-6-phosphogluconate) aldolase from *E. coli*, in *S. cerevisiae*. However, the other required enzyme, PGDH (6-phosphogluconate dehydratase), which is an iron-sulfur cluster protein, showed very low enzyme activity, when expressed in *S. cerevisiae*. The study showed the importance of establishing functional expression of iron-sulfur cluster enzymes for the integration of the ED pathway in *S. cerevisiae* [48]. More recently, the ED pathway was successfully introduced in *S. cerevisiae*. However, according to flux ratio analysis results of the engineered strain, there was little metabolic flow to this pathway. The low availability of the iron-sulfur cluster in the yeast cytosolic environment was suggested as a possible explanation for this weak ED pathway activity [49]. 

Modifying the structure and energy coupling of disaccharide metabolism and transport is also an important strategy to lower ATP generation and increase ethanol yield. *S. cerevisiae* hydrolyzes sucrose extracellularly and takes up glucose and fructose by facilitated diffusion. When this mechanism was replaced by sucrose uptake via proton cotransport and intracellular hydrolysis, the ATP required for subsequent proton extrusion reduced the anaerobic ATP yield of sucrose from 4 to 3 [50]. Basso et al. [17] achieved 4% increase in *S. cerevisiae* ethanol yield, by engineering the promoter and 5′ coding sequences of *SUC2* gene that resulted in 94% cytosolic localization of invertase. Subsequently, evolutionary engineering was applied to further increase the sucrose-uptake affinity and the ethanol yield of the engineered *S. cerevisiae* strain [17].

### 3.2. Sustainable Reduction of Glycerol Formation

In addition to biomass, glycerol is another primary by-product of first-generation bioethanol production, where approximately 5% of the sugar feedstock is converted to glycerol during industrial bioethanol fermentation [51]. Under anaerobic conditions, glycerol synthesis compensates for the depletion of NAD^+^ by re-oxidizing excess NADH from growth reactions [52]. Glycerol 3-phosphate dehydrogenase is a key enzyme for glycerol production which is encoded by *GPD1* and *GPD2* genes [53]. Fine-tuning of *GPD1* and *GPD2* expression without disrupting the regeneration of NAD^+^ can increase the ethanol yield. An increase of up to 5% was achieved in *S. cerevisiae* ethanol yield by introducing lower-strength TEF1 promoters to *GPD1* and *GPD2* genes [54]. In a later study, 61% reduction in glycerol yield and 7% increase in ethanol yield were achieved in *S. cerevisiae,* by engineering the promoter of *GPD1* in a *gpd2*Δ background [55]. To provide an alternative redox sink to glycerol synthesis, alternative pathways were introduced in a *S. cerevisiae gpd1*Δ*gpd2*Δ strain that could oxidize excess NADH by producing sorbitol and propane-1,2-diol [56]. Zhang et al. [57] decreased glycerol yield and increased theoretical maximum ethanol yield of *S. cerevisiae* by expressing *Bacillus cereus gapN* gene (non-phosphorylating NADP^+^-dependent glyceraldehyde-3-phosphate dehydrogenase), *E. coli frdA* gene (NAD^+^-dependent fumarate reductase) and *mhpF* gene (acetylating NAD^+^-dependent acetaldehyde dehydrogenase) independently [57]. Alternatively, 10% higher ethanol yield and 38% lower glycerol yield were achieved in *S. cerevisiae*, compared to the wild-type strain, by substituting normal NADPH-consuming synthesis of glutamate from ammonium and 2-oxoglutarate with the overexpression of *GLN1* encoding glutamine synthetase, *GLT1* encoding glutamate synthase, and deletion of *GDH1* encoding NADPH-dependent glutamate dehydrogenase [58]. More recently, 90% decrease in glycerol production and 15% increase in ethanol yield on sugar were achieved in *S. cerevisiae*, compared to the reference strain, by deletion of *GPD2* and heterologous expression of Calvin-cycle enzymes PRK (phosphoribulokinase) and RuBisCO (ribulose-1,5-bisphosphate carboxylase/oxygenase), to enable the use of CO_2_ as an alternative electron acceptor for the reoxidation of NADH [59]. 

The main disadvantage of a decrease in glycerol production is the reduction in osmotolerance and overall viability [60]. In a previous study by Guo et al. [61], 48.7% lower glycerol yield and 7.6% increased ethanol yield were obtained by the expression of the *GAPN* gene (NADP^+^-dependent glyceraldehyde-3-phosphate dehydrogenase) from *B. cereus* in a *gpd1*△ strain of *S. cerevisiae*. As the engineered strain became sensitive to osmotic stress, the *TPS1* and *TPS2* genes involved in the synthesis of trehalose, which is known to contribute to increased thermotolerance and ethanol tolerance in *S. cerevisiae* [62], were overexpressed and the resulting *S. cerevisiae* strain had improved ethanol yield, decreased glycerol production and improved osmotolerance [61]. Another study achieved similar fermentation performance at 38 °C and 30 °C by overexpressing the *TPS1* gene of *S. cerevisiae* which may reduce the energy cost for cooling of fermentation vessels [63]. Thermotolerance was achieved in *S. cerevisiae* at 41 °C by the overexpression of *RSP5* gene (encoding ubiquitin ligase) in a thermosensitive strain background [64]. The ethanol stress during industrial bioethanol production is one of the most challenging stress factors for yeast cells. By applying pooled-segregant whole-genome sequence analysis, *MKT1, SWS2*, and *APJ1* genes were found to be related to ethanol tolerance in *S. cerevisiae* [65]. Interestingly, increased ethanol tolerance (up to 14%) and yield from sugarcane molasses was achieved by the overexpression of a truncated version of the *MSN2* gene in an industrial fuel ethanol strain of *S. cerevisiae*, CAT-1, as reported recently [66]. CAT-1 and PE-2 are among the most widely used industrial *S. cerevisiae* strains in Brazilian ethanol plants, as they can efficiently compete with indigenous contaminant yeast and survive during industrial fermentations. It has been reported that in 2007–2008, PE-2 and CAT-1 were used in about 150 distilleries, corresponding to about 60% of the fuel ethanol produced in Brazil [67].

### 3.3. Prevention of Bacterial Contamination 

Bacterial contamination is also an important factor that may reduce the yield and productivity of bioethanol fermentations. Most commercial ethanol fermentation facilities regularly experience chronic and unpredictable acute bacterial infection due to continuous yeast propagation and non-sterile fermentation conditions which halts the fermentation process [68,69]. Lactic acid bacteria are the main contaminants that prevent yeast growth and ethanol production. To prevent contamination by lactic acid bacteria, first-generation bioethanol processes are usually carried out at pH values of 4–5. However, at low pH values, undissociated acetic acid (pKa = 4.76) readily diffuses across the yeast plasma membrane [69,70]. Thus, an acetate-tolerant industrial bioethanol strain of *S. cerevisiae* was developed using a rational metabolic engineering strategy, by overexpressing the *HAA1* gene. *HAA1* encodes a transcriptional activator which binds to an acetic acid-responsive element (ACRE), activating the expression of various targets, such as the membrane transporter genes *TPO2* and *TPO3.* The bioethanol production ability of the *HAA1*-overexpressing strain was not inhibited in the presence of 0.5% (*w*/*v*, pH 4.5) acetate, unlike the parental strain, when sugarcane molasses were used as the feedstock [71]. In a more recent study, yeast cell surface display technology was used to inhibit *Limosilactobacillus fermentum* strains in *S. cerevisiae* corn mash fermentation. For this purpose, *S. cerevisiae* EBY100 strain was used to anchor a recombinant peptidoglycan hydrolase, the lactobacilli phage endolysin LysKB317, with the a-agglutinin proteins Aga1p–Aga2p. The resulting recombinant *S. cerevisiae* strain expressing LysKB317 showed 83.8% decrease in bacterial cell counts, improved ethanol production and reduced levels of lactic and acetic acid [72]. Second generation bioethanol processes are known to be more prone to bacterial contamination due to longer pretreatment and fermentation times which allow lactic acid bacteria a longer time for competition with yeast strains [70]. Consequently, yeast strain development strategies such as improvement of acetic acid tolerance are also important for second-generation ethanol production, to prevent bacterial contamination.

### 3.4. Introduction and Optimization of Xylose Assimilation Pathway

**Figure 2:**
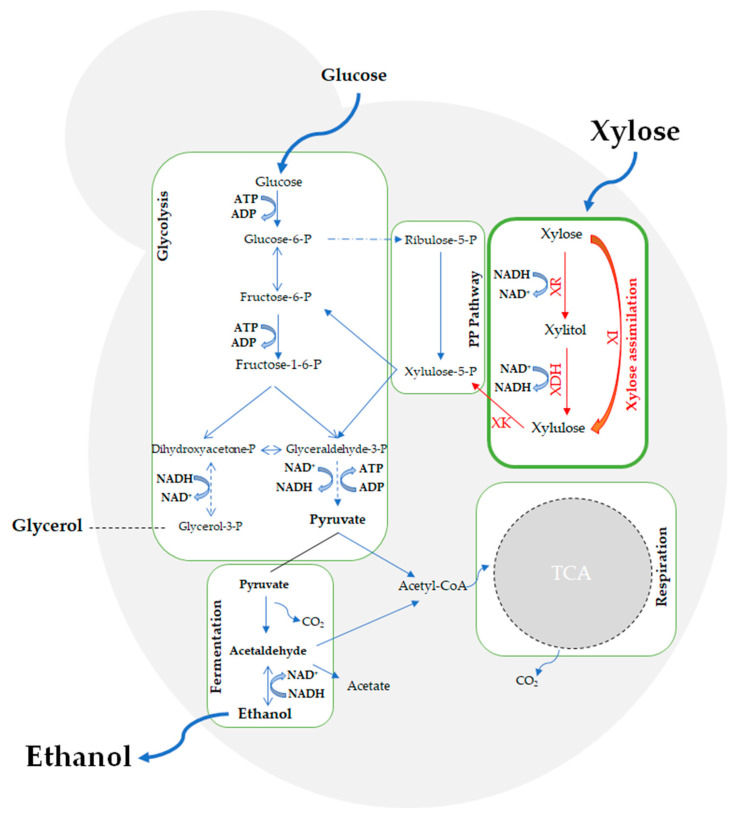
Xylose assimilation and other key metabolic pathways responsible for bioethanol production in *S. cerevisiae*. XR: Xylose Reductase, XDH: Xylose Dehydrogenase, XI: Xylose Isomerase, XK: Xylulose Kinase, PP Pathway: Pentose Phosphate Pathway.

The second-generation bioethanol production involves utilization of lignocellulosic biomass that is rich in pentose sugars, such as D-xylose and L-arabinose. *S. cerevisiae* is widely used for lignocellulosic bioethanol production due to its tolerance against ethanol, low pH, and high osmotic pressure [20]. However, as *S. cerevisiae* cannot naturally utilize pentose sugars, the introduction of specific pentose metabolic pathways to *S. cerevisiae* has been the major goal of rational metabolic engineering studies for second-generation ethanol production. For xylose assimilation, two different pathways have been introduced to *S. cerevisiae* by metabolic engineering strategies: the oxidoreductase pathway and the isomerase pathway [20,73]. The two-step oxidoreductase pathway involves xylose reductase (XR) (EC 1.1.1.307) and xylitol dehydrogenase (XDH) (EC 1.1.1.9) to convert xylose to xylitol, and then to xylulose, an intermediate that can be metabolized by *S. cerevisiae* [10,74]. The isomerase pathway, however, involves a one-step conversion of xylose to xylulose by D-xylose isomerase (XI) (EC 5.3.1.5) without cofactor requirement [10,75]. Xylulose is then further metabolized by pentose phosphate, glycolysis and fermentation pathways to produce ethanol, accomplishing xylose fermentation or assimilation in *S. cerevisiae*. Figure 2 shows the key metabolic pathways for bioethanol production in *S. cerevisiae,* including the xylose assimilation pathway that has been transferred to *S. cerevisiae* by metabolic engineering.

The introduction of the two-step oxidoreductase pathway in *S. cerevisiae* by rational metabolic engineering involves heterologous expression of genes encoding XR and XDH. However, this pathway is naturally constrained by a cofactor imbalance between the xylose reductase-using NADPH and the xylitol dehydrogenase-using NAD^+^, which causes the metabolic flux to be diverted toward undesirable products as a compensatory reaction and reduces ethanol output [76]. One of these undesirable products is xylitol. *S. cerevisiae* contains the endogenous gene *GRE3* which encodes an unspecific NADPH-dependent aldose reductase that can convert xylose to xylitol [77]. This endogenous aldose reductase, which solely utilizes NADPH as a cofactor, may exacerbate the redox imbalance in *S. cerevisiae*, leading to increased xylitol accumulation and inefficient xylose fermentation [78]. Thus, the deletion of *GRE3* gene and genetic changes that promote cofactor regeneration can reduce xylitol accumulation in *S. cerevisiae*, while increasing ethanol production. An example of this strategy is the deletion of *GRE3* gene in *S. cerevisiae* MEC1133 strain derived from the industrial strain PE-2 that is commonly used in Brazilian fuel ethanol industry. Deletion of the *GRE3* gene decreased xylitol production to undetectable levels and increased xylose consumption rate in MEC1133, leading to higher ethanol yield of 0.47 g/g of total sugars during fermentation of corn-cob hydrolysate [79]. 

There are various successful examples of introducing the two-step oxidoreductase pathway in *S. cerevisiae* by rational metabolic engineering. Li et al. [80] achieved a xylose consumption rate of 6.62 g/L/h and an ethanol yield of 0.394 at 75 g/L xylose concentration in the feed, 0.1 vvm aeration rate, 0.1/h dilution rate and 0.5 mM MgSO_4_, using an engineered and flocculent industrial *S. cerevisiae* strain KF-7 with genomic integration of *XYL1* (xylose reductase) and *XYL2* (xylitol dehydrogenase) genes from *Scheffersomyces stipitis*, *XKS1* (xylulokinase) from *S. cerevisiae*, *BGL1* (β-glucosidase) from *Aspergillus aculeatus*, and *GXS1* (glucose/xylose symporter 1) from *Candida intermedia* [80]. Another study reported 0.40 g g^−1^ cell dry weight (CDW) ethanol yield and 0.33 g g^−1^CDW h^−1^ productivity in *S. cerevisiae* by expressing *XYL1.2* (xylose reductase) from *Spathaspora passalidarum* that can also use NADPH as a cofactor, but prefers NADH; and *S. stipitis XYL2* (xylitol dehydrogenase) that can use NADH as a cofactor but prefers NADPH. The *S. cerevisiae* TMB 3044 strain used in that study had an overexpressed xylose utilization pathway and virtual absence of XR activity (Δ*gre3*) as a background [81]. Carbon dioxide is produced as a by-product during lignocellulosic ethanol production which can be recycled by introducing a synthetic reductive Pentose Phosphate Pathway (PPP) into a xylose-fermenting *S. cerevisiae* strain. Ribulose-1,5-bisphosphate carboxylase/oxygenase from *Rhodospirillum rubrum* and phosphoribulokinase from *Spinacia oleracea* were introduced into the SR8 strain of *S. cerevisiae* that harbored *XYL1* (XR), *XYL2* (XDH) and *XYL3* (xylulokinase) genes from *S. stipitis* for xylose utilization, *pho13*Δ for the upregulation of overall PPP and *ald6*Δ for the elimination of acetic acid production that is known to inhibit xylose fermentation [82]. The resulting *S. cerevisiae* strain achieved higher ethanol yield, lower yields of byproducts (xylitol and glycerol) and reduced release of carbon dioxide during xylose fermentation, compared to the control strain [82]. Carbon dioxide recycling strategy paves the way for lowering greenhouse emissions during lignocellulosic ethanol production.

The introduction of the isomerase pathway in *S. cerevisiae* by rational metabolic engineering involves heterologous expression of genes encoding xylose isomerase (XI) from various microorganisms. XI gene (*xylA*) from the bacterium *Burkholderia cenocepacia* was successfully expressed in *S. cerevisiae*. The developed strain had a 5-fold increase in xylose consumption and over 1.5-fold increase in ethanol production in a medium containing a glucose-xylose blend which resembled sugar cane bagasse hydrolysates [83]. In another study, a *S. cerevisiae* strain expressing XI from *Prevotella ruminicola* assimilated 16.95 g/L xylose and produced 6.98 g/L ethanol after 48 h of fermentation, when using xylose as the sole sugar [84]. Temer et al. [85] successfully expressed XI from the bacterium *Propionibacterium acidipropionici* with the co-expression of GroEL-GroES chaperonin complex from *E. coli* for chaperonin-assisted-folding of XI. The resulting *S. cerevisiae* strain derived from the PE-2 strain of the Brazilian fuel ethanol industry had a yield of 0.44 g ethanol/g xylose [85]. More recently, a bacterial XI gene related to the Firmicutes phylum obtained from the Brazilian goat rumen metagenomic library was expressed in *S. cerevisiae*, using codon optimization. The resulting strain achieved a higher xylose consumption rate (244 mg h^−1^) and increased ethanol yield (33 mg ethanol/g xylose), compared to the control strain [86]. 

As lignocellulosic biomass primarily consists of cellulose and hemicellulose, another valuable strategy to make lignocellulosic biomass more accessible for the target microorganisms is the heterologous expression of cellulases [87]. Yang et al. [88] introduced an expression cassette carrying a cellulase gene from *Ampullaria gigas* Spix into the *S. cerevisiae* genome [88]. The developed *S. cerevisiae* strain achieved a 23.03-fold increase in endo-1,4-β-glucanase (EG) activity, a 17-fold increase in exo-1,4-β-glucanase (CBH) activity, along with 37.7-fold higher ethanol yield, compared to the wild-type strain [88]. In another study, *CWP2* gene that codes for the major cell wall mannoprotein belonging to the GPI-protein family and plays a major role in cell wall stability and *YGP1* gene that encodes a secretory glycoprotein associated with the biogenesis of the yeast cell wall were disrupted in *S. cerevisiae* INVSc1 strain. This strain was transformed with a plasmid containing *BGL* gene (encoding β-glucosidase, a key enzyme in cellulosic production of ethanol) from *Periconia* sp. BCC 2871 fused with an anchoring protein gene, facilitating the incorporation of *BGL* gene product into the yeast cell wall. The results revealed that the disruption of *YGP1* and *CWP2* genes increased β-glucosidase activity by 63% and 24%, respectively. In addition, the *YGP1* disruptant strain produced 59% more ethanol from cellobiose, compared to the original strain [89]. 

### 3.5. Increasing Stress Tolerance

Another challenge of the second-generation bioethanol production is the inhibitory effects of toxic compounds that are released upon pretreatment of lignocellulosic feedstocks. These compounds include furfural, 5-hydroxymethyl-furfural (HMF), weak acids such as acetic acid and phenolics [90]. Thus, diverse metabolic engineering strategies have been employed to improve the tolerance of *S. cerevisiae* against these compounds and increase its ethanol yield and productivity. For example, Almeida et al. [91] developed an HMF-tolerant *S. cerevisiae* strain by overexpressing alcohol dehydrogenase genes (*ADH1* and *ADH6*) to reduce HMF to less toxic compounds. More recently, Vanmarcke et al. [92] used whole-genome transformation (WGT) method to increase HMF tolerance of an industrial *S. cerevisiae* strain. Upon extensive screening of various *S. cerevisiae* strains and non-conventional yeast species, they identified a *Candida glabrata* strain as the most HMF-tolerant one. WGT of the second-generation industrial *S. cerevisiae* strain MD4 with the genomic DNA from *C. glabrata*, followed by the selection of stable transformants in the presence of HMF revealed a novel single nucleotide polymorphism (SNP) in *AST2*^N406I^ gene that conferred improved tolerance to multiple inhibitors, including HMF and furfural [92]. Another study on furfural tolerance revealed that furfural tolerance in *S. cerevisiae* is related to the PPP genes *ZWF1*, *GND1*, *RPE1*, and *TKL1*, and overexpression of the *ZWF1* gene resulted in furfural tolerance [93]. To obtain acetic acid-resistant *S. cerevisiae*, *ADY2* gene encoding an acetate transporter was deleted which resulted in 14.7% increase in ethanol yield, in the presence of 3.6 g/L acetic acid [94]. It is important to note that immobilization of yeast cells is also an effective strategy for protection against inhibitor toxicity and to increase ethanol yield and productivity. For example, a recombinant *S. cerevisiae* GSE16-T18 strain derived from the industrial bioethanol strain Ethanol Red by inserting multiple copies of the *Clostridium phytofermentans* xylose isomerase gene was immobilized by entrapping in an alginate gel matrix. Upon immobilization, the recombinant strain could efficiently ferment xylose in the presence of very high levels of acetic acid, up to 11 g/L. Additionally, in a fixed-bed reactor and repeated batch mode using crude sugarcane bagasse hemicellulose hydrolysate, the immobilized culture achieved an ethanol yield and productivity of 0.38 g_ethanol_/g_Sugars_ and 5.7 g/L/h, respectively [95]. Another study reported on an improved *S. cerevisiae* strain that became resistant to coniferyl aldehyde, a phenolic inhibitor, by overexpressing *ATR1* and *FLR1* genes encoding putative membrane-associated transport proteins in *S. cerevisiae* [96]. Table 1 summarizes the metabolic engineering examples of *S. cerevisiae* for bioethanol production that are discussed in Section 3.1, Section 3.2, Section 3.3, Section 3.4 and Section 3.5. 

## 4. Evolutionary Engineering of Yeast for Bioethanol Production

As an inverse metabolic engineering strategy, evolutionary engineering or ALE is based on random mutagenesis and selection in repeated batch or chemostat cultivations in the presence of a selective pressure that favors a desired microbial phenotype. To increase the genetic diversity of the initial microbial population of selection, physical or chemical mutagenesis can be applied, such as UV and ethyl methanesulfonate (EMS) mutagenesis [7]. However, there are also successful examples of evolutionary engineering, in which the selection experiments were performed without prior physical and chemical mutagenesis, particularly if the selective pressure itself may have highly mutagenic characteristics, as in the case of evolutionary engineering of caffeine-resistant *S. cerevisiae*, where the high concentrations of caffeine used as the selective pressure were highly mutagenic, such that highly caffeine-resistant evolved strains were obtained without prior mutagenesis of the parental strain by UV or EMS mutagenesis [97]. Following evolutionary selection experiments, the evolved strains with the desired phenotypes are then isolated and characterized, to understand the genetic basis of their phenotypes. High-throughput screening methods and omics technologies are required for these purposes [7].

There are many successful examples of evolutionary engineering of yeast for enhanced bioethanol production (Table 2), with improved ethanol yield and productivity. Rational metabolic engineering applications may cause perturbations on specific metabolic pathways and produce rate-limiting steps on metabolism which may result in a decrease in viability and growth rate of the engineered strains. For this purpose, rational metabolic engineering and evolutionary engineering strategies are commonly combined to further increase the robustness of metabolically engineered strains [98].

Most of the evolutionary engineering studies to improve yeast for bioethanol production focus on increasing yeast growth rate and viability, decreasing by-product formation such as glycerol and biomass, improving utilization and transport of pentose sugars in lignocellulosic feedstocks for second-generation bioethanol production, and increasing tolerance to ethanol and lignocellulosic inhibitors. Examples of these evolutionary engineering studies that are discussed in Section 4.1, Section 4.2, Section 4.3 and Section 4.4 are summarized in Table 2. 

### 4.1. Increasing Growth Rate and Viability 

Increased yeast growth rate and viability are major desirable traits for industrial bioprocesses and for engineering laboratory strains for research. Short generation time is a key parameter for evolutionary engineering studies, for a time-efficient selection of evolved strains. In addition, strain improvement without a growth advantage over the background strains generally experiences challenges in the evolutionary selection procedure. Thus, increase in growth rate and viability through evolutionary engineering is a vital process both for generating robust strains and for environmental fitness [98]. 

In a previous study, evolutionary engineering was applied on *S. cerevisiae* for improved growth rate on galactose, a common sugar in nonfood crops, as the sole carbon source. Upon 62 days of selection in galactose-containing medium, three evolved strains with 24% increased specific growth rate on galactose and higher ethanol yield were isolated. The galactose metabolism of the evolved strains were similar to those of two previously obtained metabolically engineered strains with higher galactose uptake rates, however, mutations were found in the global carbon-sensing Ras/PKA pathway-related genes of the evolved strains, based on comparative whole-genome sequencing analysis results. It was suggested that the mutation found in the *RAS2* gene was responsible for the increased specific growth rate on galactose [99]. Avrahami-Moyal et al. [100] increased the specific growth rate of *S. cerevisiae* from 0.029 h^−1^ to 0.32 h^−1^, by evolutionary engineering under the selective pressure of ethanol in a turbidostat. The selection in turbidostat was performed in three steps from 6% to 8% ethanol and the growth rate increased gradually in successive steps. Comparative whole-genome sequencing of the evolved strains revealed mutations in *SSD1* and *UTH1* genes, suggesting that these mutations may be associated with the improved cell wall integrity of the evolved strains. It was concluded that the cell wall stability is an important factor in increased ethanol tolerance and growth [100].

### 4.2. Decreasing By-Product Formation

As mentioned in Section 3, biomass and glycerol are two major by-products that can decrease ethanol yield during bioethanol production. In a recent study on decreasing biomass by evolutionary engineering, heterologous hexose-proton symporters were first expressed in *S. cerevisiae.* The resulting metabolically engineered strain was then further adapted to anaerobic growth by an evolutionary engineering strategy, based on gradually decreasing oxygen levels from 100% air to 100% N_2_ in a sequential batch reactor. The final evolved strains had a 17.2% increased ethanol yield, along with a 44–47.6% decrease in biomass formation [101].

To decrease glycerol production, a common metabolic engineering strategy is to delete *GPD* genes that encode glycerol 3-phosphate dehydrogenase, a key enzyme for glycerol production [53]. Guadalupe-Medina et al. [102] applied evolutionary engineering to a *S. cerevisiae* strain with deletions of *GPD1* and *GPD2* genes and heterologous expression of *E. coli* acetaldehyde dehydrogenase gene (*mhpF*) to couple NADH reoxidation to reduce acetate to ethanol. As this metabolically engineered strain was sensitive to high sugar concentrations, it was improved by evolutionary engineering for osmotolerance, using serial batch cultivation at increasing osmotic pressure. The resulting evolved strain could grow anaerobically at high glucose concentrations (1 M), had lower glycerol production and increased ethanol yield, up to 92% of the theoretical maximum [102]. In a more recent study, to develop an improved yeast strain for efficient second-generation ethanol production, metabolic and evolutionary engineering strategies were combined: a metabolically engineered *S. cerevisiae* strain expressing xylose utilization genes was further improved by laboratory evolution on 15% wheat straw stover hydrolysate. The resulting evolved strain had increased ethanol production, higher tolerance to lignocellulosic inhibitors and 20% lower glycerol production than the reference strain [103].

### 4.3. Improving Utilization and Transport of Sugars 

As described in Section 3, a major aim of rational metabolic engineering studies for second-generation ethanol production is to introduce specific metabolic pathways in *S. cerevisiae* for the utilization of pentose sugars. Evolutionary engineering strategies are usually combined with metabolic engineering approaches to further improve such metabolically engineered strains that can utilize pentose sugars. For example, dos Santos et al. [104] first metabolically engineered a robust industrial *S. cerevisiae* strain by including genes related to pentose metabolism. They then applied evolutionary engineering to that strain for optimal xylose utilization, and the resulting evolved strains had an improved yield of 0.46 g ethanol/g xylose. Whole genome sequencing of the evolved strains revealed that *ISU1* gene encoding a scaffold protein for the assembly of iron-sulfur clusters and *SSK2* gene that is a member of MAPKKK signaling pathway are crucial for the regulation of xylose fermentation [104]. Demeke et al. [105] also combined metabolic and evolutionary engineering strategies to develop a xylose-fermenting and inhibitor-tolerant industrial *S. cerevisiae* strain. They first inserted an expression cassette with *C. phytofermentans XylA* gene encoding XI and genes encoding PPP enzymes into the genome of the industrial *S. cerevisiae* strain, Ethanol Red. Upon chemical mutagenesis, genome shuffling and selection in xylose-enriched lignocellulose hydrolysate, the metabolically engineered strain was further improved by evolutionary engineering in a complex medium with xylose, for efficient xylose fermentation. The resulting evolved strain GS1.11-26 had a maximum specific xylose consumption rate of 1.1 g/g CDW/h in synthetic medium, and 32% higher ethanol production than the parental strain, during Simultaneous Saccharification and Fermentation (SSF) of Arundo hydrolysate [105]. 

In addition to the development of pentose utilization pathways, the efficient transport of pentose sugars into *S. cerevisiae* is also crucial for second-generation bioethanol production. Thus, evolutionary engineering can also be used for improving the transport efficiency of pentose sugars: in a study by Apel et al. [106], a xylose-utilizing, metabolically engineered *S. cerevisiae* BY4742 strain with a deletion in XR gene (Δ*gre3*), and overexpressing *Piromyces sp.* XI (*pspXI)* and *XKS1* genes, was further improved by evolutionary engineering that involved sub-culturing in synthetic defined medium with 2% xylose. Comparative whole-genome sequencing of the evolved strain that was growing fastest on xylose revealed a single amino acid change in the hexose transporter gene *HXT7 (F79S)* which was associated with an increased xylose uptake rate [106].

### 4.4. Increasing Tolerance to Ethanol and Lignocellulosic Inhibitors

During industrial bioprocesses, yeast cells are faced with diverse environmental stress conditions. Thus, stress-resistance or robustness is a highly desirable trait for industrial yeasts [115]. However, as stress resistance is a multigenic and complex trait, evolutionary engineering has been a more suitable and efficient strategy than rational metabolic engineering to obtain yeast cells with high resistance against diverse stress factors. In our research group, for example, genetically stable *S. cerevisiae* cells resistant to multiple-stresses [115], oxidative stress [116], silver stress [117], starvation stress [118] and 2-phenylethanol stress [119] were successfully obtained using evolutionary engineering, and characterized by omics technologies. 

Yeast cells can also be evolved to better withstand the environmental changes and adverse conditions that occur during bioethanol production. Evolutionary engineering strategies allow yeast cells to evolve and adapt to the adverse conditions of bioethanol production, leading to increased efficiency and reduced process costs. The major challenges for yeast cells during bioethanol production are the inhibitory effects of high ethanol concentrations [120] and the presence of toxic inhibitors found in lignocellulosic hydrolysates [90,121].

Although *S. cerevisiae* is widely used for bioethanol production, high concentrations of ethanol affect cell and mitochondrial membrane, cause elevated reactive oxygen species (ROS) levels and decrease cell viability and ethanol yields [122]. Thus, increasing ethanol tolerance of *S. cerevisiae* is an important goal for successful industrial bioethanol production. A haploid laboratory strain of *S. cerevisiae*, CEN.PK 113-7D, was significantly improved by evolutionary engineering, using serial batch cultivation with gradually increasing ethanol levels. The resulting evolved strains could resist up to 12% (*v*/*v*) ethanol, a concentration at which the reference strain could not survive. They also had significantly higher ethanol productivity and titer than the reference strain during aerated fed-batch cultivation, and increased glycolytic and ribosomal protein abundance and lower respiratory activity, compared to the reference strain, based on proteomic and transcriptomic results. The study also showed that evolutionary engineeering under ethanol stress triggered diploidization of the parental strain during early steps of the selection procedure, at about 7% (*v*/*v*) ethanol stress level [107]. 

In a recent study, alternation between a weak selective pressure environment (to enhance genetic diversity) and a strong selective pressure environment (to minimize low-tolerant strains) was applied as an evolutionary engineering strategy to obtain ethanol-tolerant *S. cerevisiae* strains. Although the initial selective pressure was 18% (*v*/*v*) ethanol, after 100 generations of evolution, the evolved strains could survive 25% (*v*/*v*) ethanol for 4 h, where their parental strains could not survive even for 1 h. In addition, the evolved strains could reach higher ethanol production levels (up to 98.67 g/L) than the parental strain (78.09) g/L, when fermented in synthetic broth with 200 g/L glucose [108]. 

In second-generation bioethanol production, during the pretreatment steps of lignocellulosic feedstock such as acid hydrolysis, a significant amount of by-products are formed that have inhibitory effects on yeast cells. As mentioned in Section 3, these toxic inhibitors include furfural, 5-hydroxymethyl-furfural (HMF), phenolic compounds and weak acids such as acetic acid [80]. Strain development through evolutionary engineering is economical and has great potential to cope with lignocellulosic inhibitors, as tolerance to these compounds is genetically complex and not easy to achieve by rational metabolic engineering strategies [90,121]. 

Furfural and HMF are among the major inhibitors found in lignocellulosic hydrolysates that are produced through the dehydration of pentose or hexose sugars. A laboratory evolution study in the presence of gradually increased levels of these inhibitors showed that *S. cerevisiae* cells became significantly tolerant to furfural and HMF. Metabolite analyses of the adapted strains revealed that furfural was completely converted to furfuryl alcohol, a less toxic compound, at 30 mM without changing the ethanol yield. Similarly, HMF was fully converted to 2,5-bis-hydroxymethylfuran at 60 mM. The study showed the importance of in situ detoxification of these inhibitors by the inhibitor-tolerant, evolved yeast strains for second-generation bioethanol production [109]. In a later study, Liu and Ma [110] also investigated the transcriptomic responses of a furfural and HMF-tolerant, evolved strain, upon exposure to these inhibitors. The comparative transcriptomic analysis results revealed some key pathways such as the cell wall response, endogenous and exogenous cellular detoxification pathways and specific transcription factors like Yap1, Met4, Msn2/4 and Pdr1/3 as the main differentiated components of the inhibitor-tolerant strain, which may have a role in the complex genetics of HMF and furfural tolerance [110].

Phenolics are another major group of inhibitors found in lignocellulosic hydrolysates. One of the most toxic phenolic inhibitors found in lignocellulosic hydrolysates is coniferyl aldehyde that can reduce the performance of *S. cerevisiae* cells up to 80%, at a concentration of 1.4 mM [123]. Hacısalihoğlu et al. [111] successfully developed a highly coniferyl aldehyde-resistant *S. cerevisiae* strain by evolutionary engineering. The evolved strain could rapidly convert coniferyl aldehyde, and was also resistant to other phenolic inhibitors, including ferulic acid, vanillin and 4-hydroxybenzaldehyde. Comparative transcriptomic and genomic analysis of the evolved strain revealed major changes in protein homeostasis, cell wall integrity pathways, response to oxidative stress and oxidoreductase activity, and mutations in some genes encoding key transcription factors, such as *PDR1*, *GLN3* and *CRZ1*, which may be involved in coniferyl aldehyde resistance [111].

Weak acids are also important inhibitors found in lignocellulosic hydrolysates. As acetic acid is one of the most common weak acids, it is desirable to develop *S. cerevisiae* strains that are tolerant to acetic acid. In a recent study, evolutionary engineering strategies have been successfully employed to obtain thermo-acid tolerant and acid-tolerant *S. cerevisiae* strains. The evolved strains could grow in minimal media containing 12 g/L acetic acid at pH 4 and 30 °C, and produced high levels of ethanol, up to 29.25 ± 6 mmol/gDCW/h. Whole-genome sequencing and transcriptomic analyses revealed mutations and expression changes in key genes involved in the RAS-cAMP-PKA signaling pathway (e.g., *RAS2*) and the heat shock transcription factor (*HSF1*). Reverse engineering results indicated that *RAS2* mutation conferred acid tolerance and *HSF1* mutation conferred thermotolerance [112].

Apart from evolutionary engineering studies that focused on improving tolerance to particular inhibitors, there are also other studies where yeast strains were directly evolved in the presence of lignocellulosic hydrolysates to gain tolerance against diverse lignocellulosic inhibitors simultaneously. For example, Wallace-Salinas and Gorwa-Grauslund [113] applied evolutionary engineering to the industrial *S. cerevisiae* strain Ethanol Red by selection in the presence of 50% spruce hydrolysate and at elevated temperatures. An evolved strain which could completely reduce furfural and HMF was obtained after 280 generations of selection, and it could grow at higher temperatures (39 °C) with a high ethanol yield. The combination of inhibitor tolerance with thermotolerance is particularly advantageous for an efficient production by SSF and to reduce cooling costs in second-generation ethanol production [113]. In a later study by the same research group, whole-genome sequencing results of the evolved strain indicated the role of cell-periphery proteins (e.g., extracellular sensors such as *MTL1*) and peripheral lipids/membranes in adaptation to the combined inhibitor and temperature stresses [114]. 

## 5. Challenges of Evolutionary Engineering for Bioethanol Production

Although evolutionary engineering is generally more advantageous than rational metabolic engineering as it does not require extensive knowledge about the phenotype of interest, there are still some challenges. A major challenge of evolutionary engineering is the “trade-off” situation of the engineered strains. It is defined as the loss of another trait while a strain is being evolved for a specific trait. In evolutionary biology, the trade-off is a common concept and it is accepted as a cost of adaptation, which is an important issue in evolutionary engineering studies, particularly for industrial purposes [7,124]. In evolutionary engineering, the trade-off cannot be estimated in advance, which can result in loss of time and money. As reviewed previously [7], it is very important to perform detailed physiological and genetic analyses of the evolved strains to test if there is any trade-off in other traits, particularly the industrially important ones. Caspeta & Nielsen [125] reported that genetic adaptations of yeast to high temperatures resulted in decreased growth rate at ancestral temperatures and reduced cellular functions, while ethanol production was improved. The evolved thermotolerant strains showed decreased growth rate at temperatures below 34 °C, and metabolic rewiring of the strains caused glycerol overproduction and preadaptation to other stresses. Another trade-off situation was observed in a coniferyl aldehyde-resistant, evolved strain of *S. cerevisiae*, which became more sensitive to formic acid stress, compared to the reference strain [111]. However, it has been reported that phenotypic trade-offs which usually occur during evolutionary engineering under constant conditions can be eliminated by using dynamic cultivation procedures, such as growing a pentose-fermenting, engineered *S. cerevisiae* strain on various mixtures of glucose, xylose and arabinose, for rapid fermentation of these sugars [126,127].

Another challenge of evolutionary engineering is the long time requirement for cultivation and stress application cycles during systematic selection. It is a tedious and labor-intensive work, as the cell growth and response to the applied stress must be continuously monitored for a long time. Manual operations such as periodic passaging of the cultures during evolutionary engineering experiments may also increase the contamination risk. Partially or fully automated systems can overcome these difficulties. For example, Radek et al. [128] performed evolutionary engineering in an automated microtiter plate format and Wang et al. [129] used a microbial microdroplet culture platform for their evolutionary engineering experiments. Furthermore, low-cost automated batch or continuous culture systems for laboratory evolution have been reported in several studies [130,131,132]. The use of automated systems can also increase the number of beneficial mutations and increase the number of replicates, allowing to draw more reliable statistical conclusions about laboratory evolution [131,133,134]. However, higher costs of the automated equipment is a limitation of the automated systems. In addition, scale-up is necessary, as the automated systems are designed for laboratory-scale operations. 

## 6. Future Directions for Evolution-Based Metabolic Engineering of Yeast for Bioethanol Production

In a previous review, it has been stated that in parallel to the advancements in yeast genome sequencing, analysis and editing, evolutionary engineering has been transformed from “a simple black box strain improvement strategy” into an effective tool capable of understanding and constructing yeast cell factories [126]. Owing to recent developments in directed genome evolution strategies, strain development and modification of microbial genomes has sped up. As an inverse metabolic engineering strategy, the most challenging step of evolutionary engineering is the identification of the genetic basis that confers the selected phenotype [7]. However, rapid developments in modern and high-throughput “omics’’ technologies enable fast and accurate characterization of evolved strains, allowing the identification of the complex genetic basis of desired phenotypes. Due to the highly interconnected genotypic—phenotypic information flow, comparative analysis and understanding of cellular processes and the molecular basis of complex phenotypes are possible through multi-omics approaches [135]. 

Generation of genetic diversity by mimicking evolution is the first step of evolutionary engineering strategy. A modern technique for mutagenesis is the random base editing (rBE) system for genome evolution which involves the use of cytidine deaminase fused with DNA replication-related proteins. This system can introduce several random mutations during DNA replication and increase the mutation rate that can lead to increased genetic diversity in the starting population of selection [136]. Evolutionary engineering studies can also be combined with targeted directed evolution approaches to construct more precise mutant libraries in a shorter period of time and analyse genomic modifications faster through high-throughput-sequencing methods at population level [137]. A typical example for targeted directed evolution is the generation of transcription factor mutation libraries through global Transcription Machinery Engineering (gTME) and directional screening of target phenotype [138]. gTME and site-saturation mutagenesis on the gene encoding the transcription factor *SPT15* were recently used to increase the ethanol yield of *S. cerevisiae*. The improved strains had up to 28.5% increase in ethanol yield, and 127 amino acids were identified to have an important role in the binding efficiency of Spt15 [139]. In another recent example of gTME, the gene encoding the transcription factor *SPT8* was mutated by error-prone PCR and the generated mutant library was screened for improved ethanol production and tolerance in *S. cerevisiae*. The combined effect of two mutations in the *SPT8* gene, leading to Asn156His and Gly585Ser, were found to be associated with 8.9% higher ethanol tolerance and 10.8% increased ethanol production in the improved strains. Thus, as a targeted directed evolution approach, gTME is a modern and promising strategy that can easily improve multiple cellular traits simultaneously [140]. 

As a revolutionary gene modification technique, Clustered Regulatory Interspaced Short Palindromic Repeats (CRISPRs) and CRISPR-associated (Cas) proteins is a fast, precise and efficient targeted genome editing tool with minor disadvantages like possible off-targets. CRISPR-mediated genome editing techniques have also been applied to introduce specific changes that are expected to improve bioethanol production of *S. cerevisiae.* The gene encoding alcohol dehydrogenase (*ADH2*) was completely deleted and a frameshift mutation was introduced in the *ADH2* locus by CRISPR-Cas9 technology. The resulting *S. cerevisiae* strain had an up to 74.7% improved ethanol yield, compared to the parental strain [141]. Using CRISPR-Cas9 technology, Claes et al. [142] simultaneously expressed seven secreted heterologous lignocellulosic enzymes (endoglucanase, β-glucosidase, cellobiohydrolase I and II, xylanase, β-xylosidase and acetylxylan esterase) in a second-generation industrial *S. cerevisiae* strain AC14, without any apparent reduction in fermentation capacity. The resulting strain reached 94.5 filter paper activity units (FPU)/g CDW. Most importantly, direct conversion of lignocellulosic substrates to ethanol was achieved with that strain, without prior high-cost enzyme treatment. This enabled SSF applications with the engineered AC14 strain, leading to consolidating bioprocessing (CBP), as reported by Perez et al. [143]. CBP is a promising and low-cost, emerging strategy that reduces enzyme production, biomass hydrolysis, and sugars fermentation to a single step in a reactor. Perez et al. [143] applied CBP, using the engineered *S. cerevisiae* strain AC14 that can simultaneously secrete seven heterologous lignocellulosic enzymes and ferment xylose and glucose [142], in a synthetic medium with cellobiose, corncob xylan, glucose and xylose, and an industrial medium including the solid fraction of hydrothermally pretreated sugarcane bagasse and its liquor. They achieved 4.46 g/L/h ethanol productivity and complete hydrolysis of cellobiose and corncob xylan. The ethanol productivity in industrial medium was 1.86 g/L/h, where partial conversion of both solid and liquid fractions was observed. Another recent application of CRISPR-Cas9 technology to improve bioethanol production and reduce byproduct formation involved deletion of the *S. cerevisiae GPD2*, *FPS1*, *ADH2*, and *DLD3* genes by CRISPR-Cas9 approach. The genes were knocked-out sequentially by using targeted gRNAs for these genes, nuclease Cas9-NTC and donor DNA. The resulting strain with deletions in all four genes had 18.58% increased ethanol content and decreased contents of the byproducts glycerol, acetic acid and lactic acid by 22.32%, 8.87% and 16.82%, respectively. Transcriptomic analysis and Kyoto Encyclopedia of Genes and Genomes (KEGG) enrichment analysis results revealed that the upregulated and downregulated genes of the engineered strain were mainly enriched in carbohydrate energy metabolism, and acid metabolic pathways, respectively [144]. Owing to the use of CRISPR-Cas9-based systems in directed genome evolution strategies; deletions, activations and interferences can be generated and genome-wide libraries can be produced. Transformation of pooled gRNA plasmid libraries into Cas9-carrying strains and screening/selection of the desired phenotypes is the general workflow of CRISPR-based targeted directed genome evolution strategy [145].

For producing serial and combinatorial genomic diversity, Multiplex Automated Genome Engineering (MAGE) is a rapid directed evolution technique. It can introduce genomic mutations in many locations simultaneously, by using automated devices [146]. This system uses cDNA libraries that cover the whole genome of the microorganism and encode overexpression and knockdown mutations. These modular parts were introduced into the *S. cerevisiae* genome by using the CRISPR-Cas system and robotic automation. The successive iteration of the system and selection against acetate tolerance, glycerol utilization or isobutanol production accelerated the evolutionary selection procedure [147]. 

The genome-scale CRISPR interference (CRISPRi) system, which uses a deactivated Cas enzyme that can only bind to a target sequence and decrease its expression, can also be used to generate genome-scale knockdown libraries. Using this technique, whole genome can be targeted with an inducible library and optimized specifically to yeast spacer design rules. Owing to the use of inducible library design, dosage-sensitive and dosage-insensitive genes can be targeted similarly, unlike the previous studies. As well as library construction, the screening of individual strains can be done through amplicon sequencing, using the gRNAs like barcodes [148].

**Figure 3:**
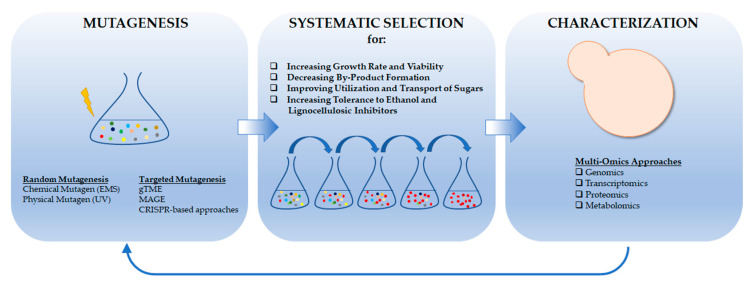
The workflow of evolutionary engineering, starting with random or targeted mutagenesis followed by systematic selection of fitter variants from this genetically diverse, initial population to obtain various improved phenotypes. Owing to high throughput omics technologies, evolved strains can be characterized in detail to understand their molecular basis.

The rapid advancements in directed genome evolution technologies reduce the limitations of evolutionary engineering and minimize the difficulties encountered on mutation generation, screening and identification of phenotype-related molecular pathways. Especially, the combination of laboratory evolution with emerging omics technologies and state-of-the-art genome editing techniques like CRISPR, will significantly accelerate research on bioethanol production. Figure 3 represents an overall summary of current and modern evolutionary engineering strategies for *S. cerevisiae* bioethanol production.

## 7. Conclusions

This review highlights the importance of metabolic and evolutionary engineering strategies for improved bioethanol production using *S. cerevisiae*. Although various rational metabolic engineering examples are also discussed in this review, a particular emphasis is given to evolutionary engineering approaches which are more advantageous than rational metabolic engineering, particularly when working with genetically complex, desirable phenotypes, as in the case of bioethanol production. This review not only gives a detailed overview of both metabolic and evolutionary engineering applications of first and second-generation bioethanol production in *S. cerevisiae*, but also includes classical gene modification techniques and directed genome evolution strategies such as CRISPR-Cas9 technology, which are used in strain improvement for bioethanol production. To our knowledge, this is the first review that emphasizes the fact that metabolic and evolutionary engineering strategies are intertwined, as many metabolically engineered strains discussed in this review were further improved for bioethanol production, by applying powerful evolutionary engineering strategies. The current limitations and future prospects of evolutionary approaches imply that the increased use of automated culture systems for evolutionary selection experiments, targeted directed evolution approaches such as gTME, combinatorial genomic diversity (MAGE) and CRISPR-based genome editing tools will all speed up evolutionary engineering research and lead to significant improvements in industrial bioethanol production by *S. cerevisiae.*

## Figures and Tables

**Table 1:** Examples of *S. cerevisiae* metabolic engineering studies for bioethanol production.

Purpose		Modification	Improvement	Reference
Lowering ATP yield	Introduction of futile cycle	Expression of *E. coli* FBPase insensitive to fructose-2,6-bisphosphate inhibition	8.8% higher bioethanol yield	[44]
	Introduction of ATPase enzyme	Heterologous expression of the F1 part of *E. coli* H^+^-ATPase enzyme	10% higher bioethanol yield	[45]
	Increasing the unspecific alkaline phosphatase activity	Overexpression of *PHO8* gene	13% higher bioethanol yield	[46]
	Introduction of Entner-Doudoroff (ED) pathway	Expression of KDPG (2-keto-3-deoxy-6-phosphogluconate) aldolase from *E. coli*	-	[48]
	Relocation of sucrose hydrolysis from the extracellular space to the cytosol	Engineering the promoter and 5′ coding sequences of *SUC2* gene	4% higher bioethanol yield	[17]
Sustainable reduction of glycerol formation	Fine-tuning of glycerol 3-phosphate dehydrogenases (GPDH)	Introducing lower-strength TEF1 promoters to *GPD1* and *GPD2* genes	5% higher bioethanol yield	[54]
	Fine-tuning of glycerol 3-phosphate dehydrogenase	Engineering the promoter of *GPD1* in a *gpd2*Δ background	7% higher bioethanol yield	[55]
	Introduction of direct cofactor regulation strategies	Expressing *B. cereus gapN* gene, *E. coli frdA* gene and *mhpF* gene independently	Increased bioethanol yield	[57]
	Engineering of ammonium assimilation	Overexpression of *GLN1* and *GLT1* genes, deletion of *GDH1* gene	10% higher bioethanol yield	[58]
	Introduction of Calvin-cycle enzymes	Deletion of *GPD2* and heterologous expression of PRK and RuBisCO	15% higher bioethanol yield	[59]
Prevention of bacterial contamination	Introduction of phage endolysin on the cell surface	Anchoring recombinant peptidoglycan hydrolase, endolysin LysKB317, by using cell surface display	83.8% decrease in bacterial cell counts	[72]
Introduction of xylose catabolism	Construction of xylose reductase-xylitol dehydrogenase (XR-XDH) pathway	Genomic integration of *XYL1* and *XYL2* genes from *S. stipitis*, *XKS1* from *S. cerevisiae*, *BGL1* from *A. aculeatus*, and *GXS1* from *C. intermedia*	Xylose consumption rate of 6.62 g/L/h and an ethanol yield of 0.394	[80]
	Construction of xylose reductase-xylitol dehydrogenase (XR-XDH) pathway	Expression of *XYL1.2* from *S. passalidarum* and *XYL2* from *S. stipitis*	0.40 g g^−1^ CDW ethanol yield and 0.33 g g^−1^ CDW h^−1^ productivity	[81]
	Construction of xylose isomerase (XI) pathway	Expression of XI gene (xylA) from the bacterium *B. cenocepacia*	5-fold increase in xylose consumption and over 1.5-fold increase in ethanol production	[83]
	Construction of xylose isomerase (XI) pathway	Expression of XI from the bacterium *P. acidipropionici* with the co-expression of GroEL-GroES chaperonin complex from *E. coli*	Yield of 0.44 g ethanol/g xylose	[85]
Improving xylose catabolism	Lowering xylitol production	Deletion of *GRE3* gene in *S. cerevisiae*	Increased ethanol yield of 0.47 g/g of total sugars during fermentation of corn-cob hydrolysate	[79]
	Introduction of a synthetic reductive PPP for carbon dioxide recycling	Expression of RuBisCO and PRK enzymes in a *S. cerevisiae* strain harboring the XR/XDH pathway	Increased ethanol yield and reduced release of carbon dioxide	[82]
Increasing accessibility of lignocellulosic biomass	Conversion of cellulose into glucose	Expression of cassette carrying a cellulase gene from *A. gigas* Spix	37.7-fold higher ethanol yield	[88]
Increasing stress tolerance	Increasing osmotolerance	Overexpression of *TPS1* and *TPS2* genes in *S. cerevisiae*	Increased ethanol yield and osmotolerance, decreased glycerol production	[61]
	Increasing thermotolerance	Overexpression of *RSP5* gene	Thermotolerance at 41 °C and ability to tolerate higher temperatures.	[64]
	Increasing ethanol tolerance	Overexpression of a truncated version of the *MSN2* gene in an industrial fuel ethanol strain	Increased ethanol tolerance (up to 14%)	[66]
	Increasing acetate tolerance	Overexpression of *HAA1* gene	The addition of acetate at 0.5% (*w*/*v*, pH 4.5) does not inhibit ethanol production	[71]
	Increasing HMF tolerance	Overexpression of *ADH1* and *ADH6* genes	Higher specific ethanol productivity in the presence of HMF	[91]
	Increasing acetic acid tolerance	Deletion of *ADY2* gene	14.7% increase in ethanol yield, in the presence of 3.6 g/L acetic acid	[94]
	Increasing coniferyl aldehyde tolerance	Overexpression of *ATR1* and *FLR1* genes	Increased coniferyl aldehyde tolerance	[96]

**Table 2:** Evolutionary engineering examples of *S. cerevisiae* for bioethanol production.

Purpose	Modification	Improvement and/or the Associated Mutations/Changes Detected		Reference
Increasing Growth Rate and Viability	Faster growth and galactose utilization	*RAS2* mutation detected	24% increased specific growth rate on galactose and higher ethanol yield	[99]
	Improved growth rate under ethanol stress	*SSD1* and *UTH1* mutations detected	Increased specific growth rate from 0.029 h^−1^ to 0.32 h^−1^ at 8% (*v*/*v*) ethanol	[100]
Decreasing By-Product Formation	Decreased biomass formation	Replacement of diffusion mediated hexose transporters with a proton-coupled transport system	44–47.6% decreased biomass production and 17.2% increased ethanol yield	[101]
	Decreased glycerol production	Evolutionary engineering of a *gpd1*Δ *and gpd2*Δ *S. cerevisiae* strain expressing *mhpF* from *E. coli* for osmotolerance revealed the mutation *mhpF ^D38N^*	Increased ethanol yield from 79% (reference) to 92%, and lower glycerol production (0.64 g/L)	[102]
	Decreased glycerol production	Evolutionary engineering in 15% wheat straw hydrolysate of an industrial yeast strain incorporating xylose genes	63.1% ethanol yield from cellulose and xylose, and 20% lower glycerol production	[103]
Improving Utilization and Transport of Sugars	Improved xylose utilization	*ISU1* and *SSK2* mutations detected	Improved yield of 0.46 g ethanol/xylose	[104]
	Improved xylose utilization	Evolutionary engineering of a *S. cerevisiae* strain expressing *C. phytofermentans XylA* gene encoding XI and genes encoding PPP enzymes	Improved maximum specific xylose consumption rate of 1.1 g/g CDW/h in synthetic medium, and 32% higher ethanol production	[105]
	Improved xylose utilization	*HXT7* mutation detected	Improved xylose uptake rate (V_max_ = 186.4 nmol min^−1^mg^−1^)	[106]
Increasing Tolerance to Ethanol and Lignocellulosic Inhibitors	Increased ethanol resistance	Triggering of diploidization	Resistance against 12% (*v*/*v*) ethanol stress	[107]
	Increased ethanol resistance	Increased glucose uptake rate and decreased lag phase	Resistance against 25% ethanol for 4 h	[108]
	Increased Furfural and HMF resistance	Transcriptomic changes associated with Yap1, Met4, Msn2/4 and Pdr1/3 transcription factors detected	30 mM furfural and 60 mM HMF resistance without loss of ethanol yield	[109,110]
	Increased Coniferyl aldehyde resistance	*PDR1*, *GLN3* and *CRZ1* mutations detected	Resistance against 2 mM coniferyl aldehyde	[111]
	Increased Thermo-acid tolerance	*RAS2* and *HSF1* mutations detected	Tolerance to 12 g/L acetate at pH 4 and 30 °C	[112]
	Increased thermotolerance	*MTL1*, *FLO9/FLO11*, and *CYC3* gene mutations detected	Furfural and HMF tolerance under thermal stress (39 °C)	[113,114]

## Data Availability

Data sharing not applicable. (No new data were created or analyzed in this study. Data sharing is not applicable to this review article.)
